# Overcoming political risk in developing economies through non-local debt

**DOI:** 10.1057/s42214-022-00137-w

**Published:** 2022-05-03

**Authors:** Jakob Müllner, Sinziana Dorobantu

**Affiliations:** 1 Department of Global Business & Trade, Vienna University of Economics and Business (WU-Vienna), Welthandelsplatz 1, 1020 Vienna, Austria; 2 Department of Management & Organization, New York University (NYU), Leonard N. Stern School of Business, 40 West Fourth Street, 707, New York, NY 10012 USA

**Keywords:** political risk, coalitions, cooperation, syndication, power dependence theory, institutional theory, nonmarket strategy, intergovernmental organizations, supranational institutions, infrastructure investments

## Abstract

Infrastructure investments are essential in generating sustainable development but also involve extensive political risk and potentially unreliable local partners. We study how banks financing such investments use syndication with non-local partners to pool economic leverage from trade, investment, and FDI. Using 5928 of the world’s largest infrastructure and energy projects in 160 countries between 2000 and 2013, we show that banks pool economic leverage from banks with dominant economic ties to the host country and from supranational institutions. Our findings contribute three distinct elements to the nonmarket strategy literature. First, they highlight the strategic value of macro-economic dependencies in the management of political risk. Second, our study positions non-local alliances as an alternative to alliance partners in the host-country context. Third, our study is the first in IB to acknowledge the value of debt-side pooling of leverage. From a practical and policy point of view, our findings suggest that practitioners and policymakers should strive to improve the efficiency of debt syndication across borders as a means of mitigating political risk and encouraging infrastructure investment.

**Supplementary Information:**

The online version contains supplementary material available at 10.1057/s42214-022-00137-w.

## INTRODUCTION

Political uncertainty in a host-country location, and the bargaining process between foreign multinationals and local sovereigns, has been at the center of IB inquiry since its inception (Vernon, 1977, 1980). Managers overseeing foreign investments have always had to balance operational decisions with strategies to protect their investments from the risk that political and social stakeholders in the host country will take advantage of their primacy in the local environment to appropriate value from the multinational investment. Social and political upheaval that directly or indirectly targets the multinational investment, government-mandated policy changes with negative implications for the project’s cash flows, and explicit demands from nonmarket stakeholders (e.g., local communities and non-governmental organizations) combine to create increasingly difficult institutional environments for multinational investments. To manage such social and political risks – in short, socio-political risk – multinational firms seek to enhance their legitimacy and influence by partnering with local firms (Brouthers & Hennart, 2007, provide a review; see also Delios & Henisz, 2000; Fabrizio, 2012; Meyer et al., 2009; Oxley, 1999) and by forming local political connections in the target country (Faccio, 2010; Siegel, 2007; Xin & Pearce, 1996).

Local partners, however, are themselves embedded in the local political landscape and influenced by policymakers. As the political environment becomes more volatile and partisan shifts become more likely, the probability that local partners become liabilities in dealing with a new group of unconstrained policymakers increases. Because of this unreliability of local partners (Siegel, 2007), multinational firms also invest heavily in non-local partnerships that provide political leverage over the host-country stakeholders. For instance, the development of the world’s largest infrastructure and energy projects has frequently involved partnerships with multinational firms from different countries and the participation of large intergovernmental organizations such as the World Bank, the European Bank for Reconstruction and Development (EBRD), and the Inter-American Development Bank (IBRD). Yet, in contrast to the growing number of studies emphasizing the importance of *local* partnerships to manage political risk in the host country (see Dorobantu et al., 2016, for a review), attention to the formation of strategic alliances with *non-local* partners that can provide political influence has been surprisingly sparse.

We argue in this paper that firms investing in countries with high socio-political risk are likely to rely on an assortment of non-local partners to protect their investment by reducing the likelihood that local stakeholders will demand renegotiation of the distribution of value specified in the initial contractual terms. We rely on a novel empirical setting – the development of large-scale infrastructure and energy projects through a form of financing known as “project finance” (PF; Esty & Sesia, 2010) – to show that investments in countries with high socio-political risk are more likely to be backed by banks from countries having political leverage over the host country, and more likely to involve the participation of intergovernmental organizations. Under the terms of PF loans, so-called non-recourse lending, banks assume extensive political risk because if local host governments take actions that threaten the repayment of the loan, there are no means recovering the capital outside of the project and its host-government jurisdiction. Using data on 5928 large-scale infrastructure and energy projects in 160 countries, jointly financed by 2647 banks between 2000 and 2013, we show that loan arrangers are more likely to form syndicates with high political leverage when they invest in locations with high levels of socio-political risk.

Our theoretical argument begins with insights from research on “macrostructure” (Rangan & Sengul, 2009) that suggests that bilateral, country-level macro-economic interdependencies lie in the background of firm-level foreign investment decisions. Trade relations, foreign direct investment (FDI), and development-aid flows create economic interdependencies between countries, which further influence economic relations between them (Lake, 2011; Milner, 1999). We propose that these broader economic relations between a firm’s home country and the host country where it is investing are particularly important considerations when the investment is in countries with high socio-political risk as they serve as socio-economic constraints on local political opportunism. In countries of political uncertainty, weak formal institutions do not prevent (and may even incentivize) opportunistic behavior by local actors, who are themselves uncertain about the future in a rapidly changing social and political landscape. As a result, multilateral investments can easily get entangled in a web of local controversies around the distribution of value generated by the investment and become dependent on local stakeholders to resolve these disputes without appropriating additional value from the firm. We argue that when facing high socio-political risk in the host country, a firm is more likely to form non-local partnerships with firms from countries with high political leverage to rebalance the dependence relations (Emerson, 1962; Gargiulo, 1993; Pfeffer & Salancik, 1978) between the firm and its local stakeholders. Such non-local coalitions with partners from economically influential countries allow MNEs to fill institutional voids in the host countries and invest despite high political uncertainty.

We highlight two important differences between local and non-local partners. First, local partners are locally embedded and therefore vulnerable to changes in the local socio-political landscape, whereas non-local partners are largely immune to such changes. The embeddedness of local partners is an important source of influence, but only in countries with stable and durable political regimes. Political events (such as elections, coups, or even peaceful regime transitions) that shift power from one set of political actors and their socio-political allies to another can quickly turn local partnerships from assets into liabilities (Fisman, 2001; Siegel, 2007). For non-local partners, the political leverage they contribute to the interorganizational partnership is *not* contingent on a specific distribution of power among local stakeholders and is therefore impervious to local events. Second, the political leverage provided by non-local partners is anchored in the broader macrostructure of bilateral relations between countries and is thus unavailable to local partners. Moreover, because bilateral relations between countries are a “sum” of a myriad of bilateral exchanges, they tend to be stable over time (Dicken, 2015) and therefore to be a source of long-term leverage over local stakeholders.

The Hamaca oil field in the Orinoco belt of Venezuela is one of the most publicized examples from our data. It is also very useful in highlighting the pooling of political leverage by the lead arranging banks. The financing of the $3.5 billion project brought together a syndicate of ten banks from seven countries on three continents. At the time of the project’s financing in 2001, Venezuela was perceived as a location with moderate-to-high local risk, ranking in the 60th percentile of the Economist Intelligence Unit’s country risk ratings. The oil companies from the US spearheading the project (ConocoPhillips, Texaco) reached out to the Royal Bank of Scotland and BNP Paribas from France to arrange a loan. These banks formed a syndicate that included ING Bank, from the Netherlands (at the time the second-largest investor in Venezuela); WestLB and Bayrische Landesbank, from Germany; Barclays, from the UK; and Bank of Tokyo-Mitsubishi, from Japan. In addition, they obtained guarantees facilitated by export–import banks in the US and Canada. Together, these banks amassed considerable economic and political leverage (their home countries accounted for over 70% of FDI and over 70% of foreign aid granted to Venezuela) to deter the Venezuelan government from altering the project terms and thus jeopardizing the repayment of the loan (Müllner & Puck, 2018).

Our study provides three important contributions to international business literature. First and foremost, we highlight theoretically and show empirically that supranational inter-country dependence relationships are boundary conditions of local political opportunism. Therein, we add an overlooked element to the literature on nonmarket strategy (Sun et al., 2021) and stakeholder management (Devinney et al., 2013). We show that firms facing high levels of political risk seek to protect their investments by embedding a project in the broader macrostructure of bilateral economic and political relations (Alcacer & Ingram, 2013; Rangan & Sengul, 2009) using interorganizational partnerships with other multinationals and with intergovernmental organizations.

Second, we contribute to the literature on alliances and political risk (Dorobantu et al., 2020; Vasudeva et al., 2012; Ahuja et al., 2011; Brass et al., 2004) highlighting that non-local partnerships with partners from countries with high economic leverage can serve as valuable substitutes for often-studied alliances with local partners in the host country. In the presence of political uncertainty, populist tendencies, or partisan rifts in the host country, local partners are often unreliable and can potentially become a liability if there is a change in government (Devinney & Hartwell, 2019). In such cases, non-local partners with partners from countries with extensive influence in the host country can serve as a more reliable substitute. We also argue that supranational organizations can serve as non-local leverage and a potential substitute for leverage from indirect economic dependence. Thus, we highlight the importance of supranational institutions like bilateral trade agreements, international arbitration, or multinational organizations that have been discussed in recent IB literature ((Doh, Rodrigues, Saka-Helmhout, and Makhija, 2017; Jandhyala & Weiner, 2014; Pinkham & Peng, 2017).

Third and finally, our study contributes a novel, debt-side, perspective to the nonmarket strategy literature in IB. While there has been some recognition of the importance of creditors in constraining governments’ opportunism in the fields of economics and finance (Dorobantu & Müllner, 2019), IB literature has not included these important actors in their theorizing or empirical analysis. We argue that – in some settings like PF – creditors are in a better position to constrain sovereigns than equity-side partners such as joint-venture partners because of the high degree of coordination and efficiency of financial markets on the one hand and the relatively easy organization of partners contributions to large banking syndicates. As such, debt syndicates can pool economic leverage more efficiently than equity partners.

### Macrostructure and the Political Leverage of Interorganizational Partnerships

A longstanding research stream in strategic management and IB has focused on the local institutional environment as a source of risk for multinational investments. Multiple studies have examined the effects of local risk resulting from weak institutional environments that allow a government to unilaterally change policies in ways that negatively affect the value of realized investments and allow opportunistic local economic actors to misappropriate value (Henisz & Williamson, 1999; Henisz & Zelner, 2001; Oxley, 1997; Oxley & Sampson, 2004). At the extreme, a government can take advantage of its territorial sovereignty to fully expropriate an investor (Kobrin, 1979; Moran, 1973). More frequently, however, governments introduce policy changes (e.g. tax hikes, regulatory restrictions) that negatively affect the net present value of existing investments. Such actions occur frequently in infrastructure industries (e.g. electricity, water, and gas) where multinationals that acquired privatized utilities experienced lower-than-expected revenues after governments reneged on contractual terms and lowered or delayed increases in utility tariffs for political reasons (Dorobantu & Zelner, 2015). At the same time, a weak institutional environment (in particular, the inadequate specification and enforcement of contracting rights) also increases the risk that local exchange partners will behave opportunistically (Oxley, 1999; Williamson, 1996) and that other local stakeholders (e.g. local communities and non-governmental organizations) will demand additional redistribution of value (Henisz et al., 2014).

Multinational investments are particularly vulnerable to socio-political risk because governments have strong incentives to offer multinational firms advantageous conditions to attract their investments but also face strong incentives to renege on their commitments *ex post*. When foreign firms invest in a country, they take resources that are mobile *ex ante* and lock them into assets that are immobile *ex post*. As this happens, the bargaining power – defined as “resources controlled by one party and demanded by the other” (Kobrin, 1987: 617) – shifts from the firm to the government. This shift, which has been conceptualized as the obsolescing bargain (Kobrin, 1987; Ramamurti, 2001; Vernon, 1977; Woodhouse, 2006) or as the fundamental transformation (Teece, 1986; Williamson, 1979), highlights that even global firms are highly vulnerable to the opportunistic behavior of governmental stakeholders in weak institutional environments. Where institutions are weak, governments lack the ability to credibly commit to not interfere with private property rights (North, 1990; North & Thomas, 1976) when they face political pressures to renegotiate contractual terms after the foreign firms’ resources have been deployed.

Scholarship has long recognized that firms investing in locations with high political risk adopt a range of strategies to mitigate such risk (Vernon, 1983). The focus of empirical research, however, has been largely on the choice between local equity partnerships and wholly owned subsidiaries (Brouthers et al., 2003; Delios & Henisz, 2000). Local partnerships allow firms to gain better insight into the political environment and influence policymaking in the host country (Delios & Henisz, 2003; Hill et al., 1990). Yet, while local partners can help navigate the local political environment, provide political connections, and possibly influence the local decision-making process, their own behavior is a source of local risk (Henisz & Williamson, 1999; Oxley, 1999). In addition, local partnerships can quickly transform from assets into liabilities if the political regime changes (Fisman, 2001; Siegel, 2007; Zhu & Chung, 2014). Thus, because local partnerships are not only a source of influence but also a potential risk, firms have incentives to find alternative means of creating leverage over local social and political stakeholders.

In this paper, we examine a complementary strategy for managing local risk. Specifically, we study the formation of *non-local* interorganizational partnerships as a means of obtaining political leverage over local stakeholders. As Vernon (1983: 203) recognized many years ago, “a consortium composed of foreigners of different nationalities is ordinarily seen as reducing the risk. … [It] can be seen as a counterforce that may be able to enlist the support of a number of different governments”. The formation of such partnerships to gain political leverage over local stakeholders is akin to the process of two-step leverage proposed by Gargiulo (1993). Drawing on the theory of power-dependence relations (Emerson, 1962; see also Pfeffer, 1981), Gargiulo (1993: 1) suggests that “an actor can gain leverage on a limiting party by building a co-optive relation with a player that may control this party’s behavior, thus using two-step leverage” (see also Bae & Gargiulo, 2004).

When faced with constraining dependencies, as many firms are when investing in foreign locations, actors engage in “balancing operations” to reduce the power imbalance (Müllner & Puck, 2018; Emerson, 1962: 35). Balancing can be accomplished through avoidance of powerful actors (Katila et al., 2008), withdrawal from relationships with powerful actors (Rowley et al., 2005), integration through mergers (Casciaro & Piskorski, 2005; Rogan & Greve, 2014), or the cooptation of additional powerful actors into a coalition that can offset the power of the influential actor (Bae & Gargiulo, 2004; Gargiulo, 1993). Among these operations, Emerson (1962: 37) views coalition formation as “the one most commonly recognized as a power process” (see also Pfeffer & Salancik, 1978). In the context of multinational investments, most of these options are unavailable after the firm has invested and the power has shifted to local stakeholders and the host-country government. Avoidance was possible *ex ante* but is no longer an option *ex post*. For a firm committed to investing in a specific (nonsubstitutable) location, vertical integration is often a way to avoid or reduce dependence on local market actors (Hari et al., 2009; Abdi & Aulakh, 2012; Fabrizio, 2012; Oxley, 1999), but avoiding the dependence on the host-country government or internalizing it through integration is impossible. Thus, building coalitions that allow the firm to increase its political leverage and partially reduce the power imbalance between it and the local stakeholders becomes the only available balancing operation in this context (Dorobantu & Müllner, 2019; Müllner & Puck, 2018).

Building an international coalition with political leverage over local stakeholders involves forming partnerships with multinational firms from countries whose governments have political influence over the local government or with intergovernmental organizations whose membership affords them broad political influence. The host country (and therefore all local stakeholders in the host country) is embedded in a macrostructure of economic and political interdependencies (trade flows, foreign investment, foreign aid, migration flows) with other countries (Guler et al., 2002; Rangan & Sengul, 2009), which can be leveraged to reduce the power dependence and thus the likelihood that local stakeholders will behave opportunistically to extract additional value from the investment. As these country-level interdependencies are largely dyadic, a partnership of firms from multiple countries is likely to have greater leverage over the local government when it involves firms from the countries on which the local government is most dependent. From the perspective of a lead arranging bank assembling a syndicate of banks for a particularly high-risk host country, pooling economic and political leverage creates an incentive to invite partners from different countries into syndicates (i.e., maximize the number of countries). At the same time, agency concerns (moral hazard and free-riding) limit the number of potential banks (and countries) so that the lead arranger will prioritize selected banks from countries in a dominant economic position vis-à-vis the host country. In other words, the lead arranger will maximize the economic and political asymmetry between the syndicate as a whole and the host country.

In an in-depth comparative case study looking back at the strategies of Kennecott and Anaconda, two copper companies with large-scale investments in Chile in the middle of the 20th century (when the risk of government expropriation was very high), Moran (1973) highlights the advantages of building a broad coalition of international actors to protect an investment against expropriation. Anaconda did little to involve foreign actors and was expropriated with no compensation. Kennecott, by contrast, took several steps to protect its investment: it took a loan from the US Export-Import Bank; it ensured the loan under a USAID guarantee against expropriation; it wrote long-term contracts with customers from Europe and Asia; and it had these loans guaranteed by a consortium of European and Japanese banks. As Moran (1973: 279) argued, “These arrangements meant that Kennecott would have a general legal claim against the Chilean state in any court should the Chilean operations be expropriated, and that the Export-Import Bank, the [US] Agency for International Development (AID), and the Congress would feel the effects of any nationalization simultaneously with Kennecott. … The aim of Kennecott was to make any threat of nationalization result unavoidably in a face-to-face confrontation between the United States and the Chilean governments”. A quote from an interview with the executive vice president of Kennecott’s Chilean operations further highlights that “the aim of these arrangements [was] to ensure that nobody expropriates Kennecott without upsetting relations to customers, creditors, and governments on three continents” (Moran, 1973: 279–280). Although Kennecott’s operations were also expropriated after further changes in Chile’s political regime, the company received full compensation for its investment because international actors pressured the Chilean government to keep its contractual obligations (Moran, 1973).

As the examples of Hamaca and Kennecott illustrate, political leverage is largely implicit. It is afforded to a firm by the political and economic relationships that exist between its home country and the country where it invests. The more that country A (the project host country) relies on country B (the home country) for international trade, foreign investment, and development aid, the less likely it is that local stakeholders in country A will interfere with the investments of firms from country B. Such interference would likely trigger discussions between the two governments during which the government of country B would try to persuade the government of country A to honor its initial commitment. If escalated, such negotiations might lead to a threat of economic sanctions, withdrawal of aid, or interruption of trade and investment. But even without explicit negotiations between the two governments, interference with the investment of a firm from country B provides a strong signal to other firms from the same country and might trigger a reassessment of the extent to which country A continues to be a desirable trade partner and investment location. Taken together, the expected reactions of the government and of other firms from country B are likely to deter stakeholders in country A from interfering with investments by multinationals from country A in the first place. These dynamics therefore afford firms from country B considerably more political leverage than firms from countries on which country A is less dependent in terms of trade relations, foreign investment, and development aid. We therefore propose the following:

#### Hypothesis 1:

When investing in locations with high political risk, syndicate lead arrangers are more likely to form syndicates that bring together banks from countries having high political leverage over local stakeholders in the host country.

### Political Leverage from Intergovernmental Organizations

In addition to forming transnational partnerships to manage political risk in the host country, syndicate lead arrangers can also increase the syndicate’s political leverage by soliciting the participation of influential intergovernmental organizations such as the World Bank or the EBRD. Intergovernmental organizations are supranational forums for addressing international concerns and exchanging information about cross-border issues (Alcacer & Ingram, 2013; Jandhyala & Phene, 2015). Their broad membership and far-reaching mandates empower them to act as representatives of the international community. Consequently, their involvement provides extensive levels of political leverage to any venture.

In the context of international investments, intergovernmental organizations play a central role in protecting investments in high-risk countries. These organizations pool political leverage from their member states. The World Bank, for instance, represents 188 countries and is thus among the global actors with the highest levels of political leverage (Gamso & Nelson, 2019). In addition, intergovernmental organizations have leverage over local governments because they pool financial resources from the member states and offer them as development funds to governments under preferential financial conditions. Thus, a government is unlikely to change the terms of an investment backed by intergovernmental organizations for fear that such behavior will affect its access to future development funds.

At the same time, the multinational nature of intergovernmental organizations makes them powerful forums for diffusing information about a specific government’s opportunistic behavior, threatening repercussions by a broader community of countries. This special status allows intergovernmental organizations to provide a “political umbrella” to single projects (Hainz & Kleimeier, 2006, 2012; Sorge, 2004: 100). Woodhouse (2006) shows that contract renegotiations in international projects are more effective when intergovernmental organizations are part of the partnership. Sorge (2004) and Gatti et al. (2013) show that the participation of intergovernmental organizations decreases international loan margins by signaling lower investment risk to banks. Building on these insights, we hypothesize that intergovernmental organizations can exert direct political leverage, thereby making them attractive participants in interorganizational partnerships that invest in locations with high levels of socio-political risk.

The participation of intergovernmental organizations, however, does not come without additional costs to the syndicate. By design, intergovernmental organizations are multi-tiered institutions that require extensive and iterative bargaining between the members throughout the decision-making process. In addition, their charters mandate that they structure their activities to promote development goals and that they enforce high standards of governance as well as environmental and social responsibility. For instance, the World Bank agreed to participate in the financing of the $3.7 billion Chad–Cameroon pipeline project only after the government of Chad agreed to implement a revenue management plan that would bind it to directing most of the revenue generated by the project to poverty alleviation programs (Esty, 2003). More recent projects involving World Bank financing required that projects abide by the strict Environmental and Social Sustainability Standards (known as the “Equator Principles”) of the International Finance Corporation, the World Bank’s investment body. These added layers of requirements and negotiations often delay project development timelines and increase project costs. The additional complexity might nonetheless be worth the cost if it provides additional leverage to protect projects in high-risk countries. As Andrea Macdonald (1998: 122), a former Exxon executive, highlighted while reflecting on past experience, “Political risk associated with large-scale projects in the developing world is a reality that must be thoughtfully assessed and carefully addressed in project planning … While the involvement of multilateral [intergovernmental] institutions and other lenders adds complexity, their presence can enhance country commitment and mitigate political risk”. Building on these insights, we argue the following:

#### Hypothesis 2:

When investing in locations with high political risk, syndicate lead arrangers are more likely to form syndicates that include the participation of intergovernmental organizations.

## EMPIRICAL SETTING, DATA, AND METHODS

We examine the effect of socio-political risk on the composition of multinational partnerships using data on banking syndicates that formed to finance some of the world’s largest infrastructure projects using a specific type of financing known as project finance (PF). In 2015 alone, the total amount of PF loans exceeded $275 billion, making PF one of the most important forms of finance for large infrastructure projects (Project Finance International, 2016). Despite the global presence and economic significance of PF, only a few studies have explored the intricacies of such investments (Esty & Megginson, 2003; Sawant, 2010) or taken advantage of this empirical setting to study broader strategy and IB research questions ( Byoun et al., 2013; Vaaler et al., 2008; Byoun & Xu, 2014; Esty, 2004; James & Vaaler, 2013; Müllner, 2016).

Examining large infrastructure projects financed through PF offers multiple advantages. First, these projects are established as stand-alone organizations ((Brealey, Cooper, and Habib, 1996; Nevitt & Fabozzi, 2000) because the companies who propose them (so-called sponsors) are reluctant to assume the full risk associated with the development of particularly large and risky assets (Orr & Scott, 2008). As a result, a project company is established as a separate, legally and financially independent entity. Because of the non-recourse nature of PF lending, creditors assume high political risk. Projects are designed in a way to maximize the use of debt, with very low debt-service coverage ratios. This means that any external intervention from local political actors threatens the project’s survival. If a project fails, banks have no other securities than the location-fixed assets of the project in the foreign host country and no legal claims against equity providers. Banks commonly have no option other than to renegotiate or pursue legal actions against the host-country government. Moreover, because the project company has only one project, the strategic decisions made by project companies can be studied without distortions resulting from the combination of multiple investments on one balance sheet (Esty, 2004; James & Vaaler, 2013).

Second, such projects are financed through a large loan, structured as non-recourse debt. When banks agree to offer non-recourse debt, they accept that they cannot rely on the project sponsors’ balance sheets to secure their loans (Byoun & Xu, 2014; Hainz & Kleimeier, 2012). Instead, repayment of the loan is contingent on the project’s future cash flows. In the event of a default, the loan cannot be fully recovered from the sponsors. The banks’ risk is therefore directly tied to project performance and not to sponsors’ creditworthiness (as is the case in traditional corporate finance). As a result, banks participating in the financing of large PF projects have strong incentives to form a syndicate that can protect the investment from any interference.

Third, the location and timing of a project is determined by a sponsor without a sense of the composition of the banking partnership that might form to finance it, as the banks come together only after the project is fully defined. This allows us to directly examine how exogenous variation in socio-political risk shapes the composition of banking partnerships, without concerns that the composition of the syndicate determines the characteristics of the project or its location (Orr & Scott, 2008).

Finally, PF investments span a broad spectrum of political contexts. Specifically, roughly 40% of the projects are located in developing countries and account for a similar percentage of the global volume (Esty & Sesia, 2010; Hainz & Kleimeier, 2006). At the same time, PF is strongly concentrated in infrastructure sectors (35% in the power sector, 21% in transportation, 16% in oil and gas), in which location-specific assets are highly susceptible to local, socio-political risk. Infrastructure projects are large, capital-intensive developments that do not generate revenues until they are largely completed. This setting, which requires large upfront investments during the construction phase of the project, followed by a revenue-generating phase that requires only minimum investments in its operation and maintenance, creates strong incentives for local stakeholders to use their advantageous position in the local institutional environment to renegotiate the contractual terms of the project in their favor. For instance, a government agency operating a toll road has incentives to appropriate the revenue generated by the toll collection instead of using it to repay the initial loan, as specified in the loan contract.

### Data and Methods

Our dataset includes 7833 infrastructure investments in 160 countries that were financed by syndicates made up of 2647 banks. The data were obtained from Dealogic Projectware and the Thomson SDC Platinum database, the two most complete sources of information on PF investments (Byoun et al., 2013; Gatti, 2012). The total volume of financing in our sample amounts to $3.7 trillion, which is close to Germany’s GDP in 2015. We use the full dataset to estimate whether a project will be financed through a banking partnership that includes at least one foreign bank, and we control for this selection process when evaluating the relationship between local socio-political risk and the composition of the syndicates that financed 5928 projects in 160 countries between 2000 and 2013 (Appendix A describes the distribution of projects across countries and years).

The average project value in our sample is $509 million, and the median project value is $207 million. The largest single project in the dataset is the Ichthys liquid natural gas oil field project in the northern territory of Australia, which has a total project value of $34 billion. The sectors with the most projects are power generation (1126 projects), wind farms (661), renewable fuel (602), roads (428), and mining (326) (see Appendix B). Many of the banks providing PF loans come from the US (371 banks), the UK (170), Japan (169), South Korea (115), and Spain (111). The most active banks in the dataset in terms of number of projects are BNP Paribas, from France (874 projects); Bank of Tokyo-Mitsubishi, from Japan (686); Sumitomo Mitsui Banking Group, from Japan (649); and West LB, from Germany (646); 1112 banks participate in just one project.

We complement this information with data on the banks involved, country-level risk indicators, and data on economic flows, as we describe in detail below. Table 1 includes variable descriptions and summary statistics. Table 2 shows the correlations.

**Table 1:** Variable description and summary statistics

		Observations	Mean	SD	Min	Max
Dependent variables measuring political leverage						
(1) Trade leverage	Total trade (imports plus exports) between project host country and countries in the international banking syndicate (as a percentage of total trade of host country) minus total trade between project host country and countries in the syndicate (as a percentage of total trade of countries in the syndicate), 10-year average. (Source: UN Comtrade Database)	5928	5.96	41.69	− 556.45	360.42
(2) Export leverage	Exports of project host country to countries in the syndicate (as a percentage of total exports of host country) minus total exports of countries in the syndicate to the project host country (as a percentage of total exports of countries in the syndicate), 10-year average. (Source: UN Comtrade Database)	5928	5.26	45.90	− 636.26	431.18
(3) Import leverage	Imports in project host country from countries in the syndicate (as a percentage of total imports of host country) minus total imports in countries in the syndicate from the project host country (as a percentage of total imports of countries in the syndicate), 10-year average. (Source: UN Comtrade Database)	5928	6.64	38.60	− 446.73	407.24
(4) FDI leverage	FDI stock from countries in the syndicate to project host country (as a percentage of total FDI in project host country) minus the FDI stock from project host country to countries in the syndicate (as a percentage of total FDI in countries in the syndicate), 10-year average. (Source: UNCTAD Bilateral FDI Database)	5928	− 2.99	68.00	− 2,104	800
(5) Aid leverage	Total development aid from syndicate members’ countries into project host country (as a percentage of total aid of host country), 10-year average. (Source: AidData.org)	5928	5.85	37.79	− 483.17	994.77
(6) Intergovernmental organization	Participation of an intergovernmental organization or development bank in the syndicate. (Source: Dealogic Projectware Database)	5928	0.09	0.28	0.00	1.00
Independent variables						
(7) Socio-political risk (EIU)	Economist Intelligence Unit political risk score for project host country (*z*-standardized measure). (Source: Bureau van Dijk electronic publishing)	5737	0.00	1.00	− 1.36	3.55
(8) Socio-political risk (PRS)	ICRG Political risk index for project host country provided by the PRS group (*z*-standardized measure). (Source: The PRS Group, 2015)	5854	0.00	1.00	− 1.89	7.39
Controls						
(9) Project size	Total project amount ($ million), log values. (Source: Dealogic Projectware Database)	5924	12.24	1.34	6.58	17.34
(10) Project with offtaker	Project with offtake agreement (Source: Dealogic Projectware Database)	5928	0.16	0.37	0.00	1.00
(11) Debt-to-equity ratio	Debt-to-equity ratio reported for the project (Source: Dealogic Projectware Database)	5928	0.85	0.21	0.00	1.00
(12) Systemic risk	180-day volatility of MSCI World Barra Index by Morgan Stanley Capital International. (Source: Bloomberg)	5928	0.17	0.07	0.08	0.43
(13) Total syndicate participants	Count number of partners in the syndicate (Source: Dealogic Projectware Database)	5928	5.75	6.10	1.00	60.00
(14) No. of countries	Count number of countries in the syndicate (Source: Dealogic Projectware Database)	5921	3.04	2.54	1	20
(14) Previous ties	Average number of previous ties between partners in the project (Source: Dealogic Projectware Database)	5928	10.24	17.91	0.00	192.00
(15) Previous PF experience	Average number of previous project finances by partners (Source: Dealogic Projectware Database)	5922	3.26	2.68	0.00	17.00
(16) Banks’ local presence	Count number of subsidiaries (>50%) in host country owned by all partners. (Source: Bureau van Dijk, Orbis Database)	5928	2.06	1.49	0.00	6.45
Lead arranger	Indicator variables for 240 most active lead arrangers					
Project sector	Project sectors indicators (Source: Dealogic Projectware Database)					
Project host country	Indicator variable for host countries used in robustness checks

**Table 2:** Correlations

Variable name	(1)	(2)	(3)	(4)	(5)	(6)	(7)	(8)	(9)	(10)	(11)	(12)	(13)	(14)	(15)	(16)	(17)
(1) Trade leverage	1.00																
(2) Export leverage	0.98	1.00															
(3) Import leverage	0.97	0.91	1.00														
(4) FDI leverage	0.55	0.57	0.52	1.00													
(5) Aid leverage	0.29	0.27	0.31	0.08	1.00												
(6) Intergov. organization	0.08	0.08	0.08	0.06	0.05	1.00											
(7) Socio-political risk (EIU)	0.28	0.28	0.27	0.22	0.23	0.23	1.00										
(8) Socio-political risk (PRS)	0.19	0.19	0.17	0.16	0.19	0.19	0.85	1.00									
(9) Project size	0.11	0.09	0.12	− 0.02	0.11	0.08	0.07	0.05	1.00								
(10) Project with offtaker	− 0.03	− 0.04	− 0.02	− 0.06	0.03	− 0.02	0.03	0.03	0.08	1.00							
(11) Debt-to-equity ratio	0.01	0.01	0.01	0.00	− 0.02	− 0.07	− 0.08	− 0.07	− 0.20	− 0.08	1.00						
(12) Systemic risk	0.00	0.00	0.00	0.02	− 0.03	0.04	0.06	0.08	− 0.03	0.04	− 0.01	1.00					
(13) Total syndicate participants	0.17	0.13	0.21	− 0.04	0.23	0.03	0.09	0.06	0.59	0.08	− 0.03	− 0.06	1.00				
(14) Nr. of countries	0.18	0.14	0.22	− 0.05	0.21	0.12	0.08	0	0.53	0.12	− 0.04	− 0.09	0.81	1.00			
(15) Previous ties	0.02	0.02	0.01	− 0.01	− 0.02	− 0.09	0.00	0.05	0.14	0.06	0.01	0.09	0.02	0.01	1.00		
(16) Previous PF experience	0.02	0.02	0.02	− 0.01	0.01	− 0.11	− 0.13	− 0.16	− 0.04	0.04	0.04	− 0.05	− 0.11	0.02	0.34	1.00	
(17) Banks’ local presence	− 0.29	− 0.28	− 0.28	− 0.19	− 0.15	− 0.18	− 0.44	− 0.32	0.10	0.01	0.00	− 0.02	0.06	− 0.06	0.13	0.06	1.00

#### Dependent variables

To examine a banking syndicate’s political leverage, we construct several variables using data on bilateral trade, FDI, and bilateral aid.

*Trade Leverage* We measure bilateral trade leverage by calculating the difference between the total trade (exports plus imports) between project host country $h$ and each country $i$ represented in the banking syndicate as a percentage of project host country $h$’s total trade, and the total trade between project host country $h$ and each country $i$ represented in the banking syndicate as a percentage of project host country $i$’s total trade. Thus, our measure of trade leverage reflects the extent to which country $h$’s exports and imports are concentrated in its trading with the countries represented in the syndicate as well as the symmetry or asymmetry of this dependence (Casciaro & Piskorski, 2005; Emerson, 1962). More specifically, we calculate:

$$Trade\_Leverage=\sum_{i=1}^{s}\left(\frac{{Trade}_{ih}}{{Trade}_{h}}-\frac{{Trade}_{ih}}{{Trade}_{i}}\right)$$

where $Trade\_Leverage$ refers to the leverage of the syndicate over the host country; ${Trade}_{ih}$ refers to the level of trade between the banks’ home country $i$ and project host country $h$; ${Trade}_{h}$ refers to the total trade of project host country $h$; and ${Trade}_{i}$ refers to the total trade of country $i$. We use average trade flows over the previous 10 years obtained from the United Nations Comtrade dataset. We further differentiate between *Import leverage* and *Export leverage* (which we calculate using equivalent formulas) and show that our results are similar when using the disaggregated measures instead of the combined exports plus imports trade measure.

Interpretation of these leverage measures requires some explanation. In a dyadic (bilateral) relationship, export dependence for a given project country on a given bank’s home country is + 100 if the bank’s home country accounts for 100% of exports of the project host country *and* the bank’s home country does not export to the project host country at all. It is − 100% if the bank’s home country imports everything from the project host country. Bilateral dependencies are aggregated for all dyads in the syndicate. For example, the syndicate that came together to finance the $2.3 billion Baku to Ceyhan pipeline in Azerbaijan included 15 banks from nine countries, including the US, Japan, Netherlands, Germany, Italy, France, and the UK. Azerbaijan’s exports to these countries and its imports from these countries represent almost 100% of its exports and imports, while these countries’ exports to and imports from Azerbaijan represent only a very small share of their own exports and imports. In this case, our measure of *Trade leverage* is 112.96, indicating that the syndicate has strong political leverage over the host country. Negative values of trade leverage reflect the opposite arrangement.

*FDI Leverage* Similarly, we calculate leverage obtained through bilateral FDI by summing bilateral FDI stocks in the project host country and originating from the banks’ home countries represented in the PF syndicate as a percentage of total FDI stock in the project host country. Again, we subtract from it the total of FDI stock flows from the project host country in each of the banks’ home countries represented in the PF syndicate as a percentage of the total FDI stock in these countries. Specifically, we calculate:

$$FDI\_Leverage=\sum_{i=1}^{s}\left(\frac{{FDI}_{ih}}{{FDI}_{h}}-\frac{{FDI}_{hi}}{{FDI}_{i}}\right)$$

where $FDI\_Leverage$ refers to the leverage of the syndicate over the host country; ${FDI}_{ih}$ refers to the level of FDI stock originating from country $i$ and going to host country $h$; ${FDI}_{h}$ refers to the total level of FDI stock received by country $h$; ${FDI}_{hi}$ refers to the level of FDI stock from country $h$ to country $i$; and ${FDI}_{i}$ refers to the level of FDI stock received by country $i$. We calculate this measure using the average bilateral FDI stocks over the previous 10 years. We obtained data on bilateral FDI stocks from the United Nations Conference on Trade and Development (UNCTAD) Bilateral FDI Statistics dataset.

*Aid Leverage* Unlike trade and FDI, development aid tends to be unilateral (flowing from high-income to low-income countries). As a result, we do not account for reciprocity. We estimate the partnership’s leverage obtained through bilateral aid as the total of bilateral aid received by the host country $h$ from the countries represented in the PF syndicate as a percentage of total aid obtained by the host country $h$, as follows:

$${Aid\_Leverage}_{h}=\sum_{i=1}^{s}\left(\frac{{Aid}_{ih}}{{Aid}_{h}}\right)$$

We calculate this measure as the average of bilateral aid flows over the previous 10 years to ensure that outlier values of aid due to unusual events (e.g., epidemics, natural disasters) do not influence our results. We obtained bilateral aid data from AidData 3.0, a research lab that provides development finance data (www.AidData.org).

*Intergovernmental Organization* A syndicate can also increase its political leverage through the participation of an intergovernmental organization. Our hypothesis suggests that such participation confers political and economic leverage over the host country. We measure the participation of an intergovernmental organization in the project using a dummy variable. The most frequent intergovernmental organizations participating in the financing of large projects are the International Finance Corporation, the EBRD, the Asian Development Bank, and the Inter-American Development Bank.

#### Independent variables

*Socio-political Risk* We use two different measures to capture the effect of socio-political risk, the key independent variable in the analysis. First, we rely on a measure of socio-political risk constructed by the Economist Intelligence Unit (EIU) to capture a range of factors relating to “political stability and effectiveness that could affect a country’s ability and/or commitment to service its debt obligations and/or cause turbulence in foreign exchange markets,” such as external conflict, social unrest, electoral cycle, institutional effectiveness, and corruption (EIU, 2015). The EIU index is built by country experts who pay regular visits to the countries they cover and maintain large networks of local contacts. The index is used widely in the banking sector (the empirical setting in this paper), by asset managers, and in treasury departments. Second, we use the International Country Risk Guide (ICRG) score provided by the Political Risk Service (PRS) as an alternative measure. Their political risk rating includes 11 weighted variables: government stability, socio-economic conditions, internal conflict, external conflict, corruption, military in politics, religious tensions, law and order, ethnic tensions, democratic accountability, and bureaucratic quality (PRS, 2015).

We rely on the EIU and ICRG measures for assessing the degree of socio-political risk in a country in a given year because of the breadth of their coverage. Unlike other measures that focus on the rule of law (e.g., La Porta et al., 1998), political constraints (Henisz, 2000), or electoral competition (Marshall & Jaggers, 2000) and that capture only the checks and balances placed on governments to ensure that they do not interfere with contract enforcement or change policies without due process, the EIU and ICRG measures also reflect the degree of social tension within society (for socio-economic, religious, or ethnic reasons) and the possibility of internal or external military conflict, all of which could affect large infrastructure projects and the repayment of the loans provided for their development. In our robustness analyses, we test the sensitivity of our results using the Polcon measure of political constraints constructed by Henisz (2000) and the Polity measure of political competition (Marshall & Jaggers, 2000). We standardize all measures of socio-political risk.

*Project-Level Controls* We include a number of project-level and syndicate-level controls in our analysis. At the project level, we control for the size of the project being financed, as this might influence the composition of the banking syndicate. The measure *project size* is the log of the project’s value (in $US millions) reported in the Dealogic database. We also create a dummy to control for projects with recorded *offtake agreements*. Such agreements guarantee a purchaser (so-called offtaker) for the project’s output (e.g., the electricity generated by a power plant) and have been found to significantly reduce a project’s financial risk (James & Vaaler, 2013). Further, we control for the project’s capital structure using the *debt-to-equity ratio* reported in the Dealogic database. We also include in our analysis a measure of *systemic risk* to capture the degree of volatility in financial markets during the time the project was financed. We measure systemic risk using the 180-day volatility from the MSCI World Barra Index published by Morgan Stanley Capital International and, alternatively (in our sensitivity analyses), using a financial crisis dummy (coded 1 between September 10, 2008, and September 10, 2010) and year fixed effects.

*Syndicate-Level Controls* We also control for a number of factors at the syndicate level. First, we control for the *number of partners* in the syndicate and the *number of countries* included in the syndicate. The project loan is provided by 5.75 partners, on average, and 10% of projects include more than 12 partners. Second, we control for the number of *previous ties* between banks, as these are likely to influence the formation of additional partnerships (Gulati, 1998). We measure previous ties as the number of projects sponsored in the past (not including the current year) by at least two of the banks in the current syndicate. Among all the bank dyads in our data, 25% appear in the data five or more times, 35% appear one to four times, and 40% are new partnerships. At the high end, BNP Paribas and Bank of Tokyo-Mitsubishi have worked together to finance 96 different projects around the world (representing the maximum number of previous ties in our data). Third, we control for banks’ *previous experience* in financing PF projects, as this may also affect the sensitivity of partnerships to socio-political risk. Firms learn through international experience (Barkema et al., 1996; Chang, 1995; Johanson & Vahlne, 1977; Perkins, 2014) and build political capabilities and relational capital when investing abroad (Hillman & Hitt, 1999), which they can leverage to mitigate local political risk (Barkema & Vermeulen, 1998; Delios & Henisz, 2000, 2003). Fourth, we also control for the *banks’ local presence* in the host country by including the number of local subsidiaries that are majority-owned by the syndicate partners. We constructed this variable using information on each of the bank’s global subsidiary networks from Bureau van Dijk’s Orbis database.

Finally, the formation of a banking syndicate is largely driven by the *lead arrangers* for the syndicate. Banks that serve in these roles may differ systematically in their propensity to form global syndicates with partners from certain countries. To account for such heterogeneity, we identify the lead arrangers for each syndicate, and we include in the analysis 240 indicator variables representing the banks that have served as lead arrangers for more than ten projects across our sample. This cut-off point identifies the most active lead arrangers and allows us to control for the potential clustering of data while keeping our empirical estimation computationally feasible. Similarly, we control for heterogeneity among sectors by including sector-level fixed effects.

## RESULTS

We argue that syndicates mitigate high socio-political risk in the project host country by bringing together banks from countries with political leverage over stakeholders in the host country. We examine this relationship by estimating a series of regressions using different measures of political leverage as dependent variables and local socio-political risk as the main independent variable. The results are estimated using multilevel random intercept models (Skrondal & Rabe-Hesketh, 2004). The unit of analysis is the project; country-level random intercepts account for the hierarchical structure of the data (i.e., multiple projects within a country); lead-arranger fixed effects account for heterogeneity among syndicate lead arrangers; and sector fixed effects account for sector-level differences between projects. Results hold if we add random slopes to random intercepts.

One concern in analyzing the relationship between socio-political risk in the project host country and the decision to seek financing outside the project country is influenced by the level of development of domestic financial markets and the availability of domestic credit. To address this concern, we first run a selection model (Heckman, 1979) that estimates whether a project investment will be financed through an international (rather than domestic) bank syndicate. We evaluate this decision using data on local financial market development measured according to the World Bank Global Financial Development Report (Čihák et al., 2012), host-country GDP, and local political risk, all of which affect the likelihood of foreign project financing. We also include in the selection equation project-specific variables: project size, presence of an offtaker, debt-to-equity ratio, and year fixed effects (for a similar approach, see Lavie & Miller, 2008; Zhu & Chung, 2014). Using the results of our selection model (not shown), we predict the probability of a non-domestic international banking syndicate and use the inverse Mills ratio from this analysis in all the models we present below. In our sensitivity analysis, we show that the results are unchanged when we exclude the selection correction from our specifications.

In Table 3, we present the results using the EIU measure of socio-political risk. We repeat the estimations using the PRS measure of socio-political risk in Table 4. In models (1) through (5) of Table 3, and in models (7) through (11) of Table 4, respectively, we find very strong support for all measures of political leverage (H1). In Table 3, trade leverage (*β* = 7.465;* p* = 0.000), export leverage (*β* = 7.540; *p* = 0.000), import leverage (*β* = 7.406; *p* = 0.000), FDI leverage (*β* = 12.34; *p* = 0.000), and aid leverage (*β* = 11.49; *p* = 0.000) are all positive and highly significant. *T*-statistics range from 4.35 for export leverage to 8.12 for aid leverage. This provides strong support for our Hypothesis 1 that lead arrangers actively include in the syndicate those banks from countries having strong political leverage. Using the PRS measure in Table 4, we observe coefficients with a very similar pattern. Trade leverage (*β* = 5.813; *p* = 0.000), export leverage (*β* = 6.754; *p* = 0.000), import leverage (*β* = 4.689; *p* = 0.000), FDI leverage (*β* = 6.078; *p* = 0.004), and aid leverage (*β* = 7.832; *p* = 0.000) are all significant, with T-statistics ranging between 2.88 for FDI leverage and 5.75 for aid leverage.

**Table 3:** Random intercept estimates (including syndicate lead-arranger fixed effects)

	Political leverage (H1)					IGO (H2)
	(1)	(2)	(3)	(4)	(5)	(6)
	Trade leverage	Export leverage	Import leverage	FDI leverage	Aid leverage	Intergov. organization
Socio-political risk (EIU)	7.465***	7.540***	7.406***	12.34***	11.49***	0.317***
	**(4.74)**	**(4.35)**	**(5.01)**	**(5.30)**	**(8.12)**	**(3.97)**
Project size	13.06***	14.64***	11.09***	16.05***	0.944	0.325***
	(18.73)	(18.59)	(17.33)	(14.99)	(1.18)	(4.95)
Project with offtaker	7.704***	8.848***	6.486***	7.068***	− 0.0928	0.0314
	(5.94)	(6.03)	(5.46)	(3.54)	(− 0.06)	(0.24)
Debt-to-equity ratio	18.13***	20.72***	15.20***	21.84***	− 0.506	0.121
	(7.15)	(7.23)	(6.54)	(5.59)	(− 0.17)	(0.48)
Systemic risk	− 24.98***	− 29.21***	− 19.80***	− 31.82***	− 6.788	0.915
	(− 4.15)	(− 4.29)	(− 3.58)	(− 3.43)	(− 0.96)	(1.53)
Total syndicate participants	0.363*	0.148	0.633***	− 1.165***	1.608***	− 0.0820***
	(2.40)	(0.86)	(4.57)	(− 5.01)	(9.10)	(− 6.04)
Previous ties	− 0.0384	− 0.0427	− 0.0349	− 0.121**	− 0.0868**	− 0.0108*
	(− 1.39)	(− 1.37)	(− 1.38)	(− 2.86)	(− 2.69)	(− 2.52)
Previous PF experience	− 0.203	− 0.293	− 0.107	− 0.300	0.723**	− 0.0281
	(− 0.85)	(− 1.09)	(− 0.49)	(− 0.82)	(2.60)	(− 1.07)
Banks’ local presence	− 1.208**	− 1.453**	− 0.989*	− 1.192	− 0.962	− 0.0354
	(− 2.67)	(− 2.85)	(− 2.38)	(− 1.72)	(− 1.86)	(− 0.73)
Nr. of countries	− 1.157**	− 1.431***	− 0.926**	− 2.382***	− 0.860	0.224***
	(− 3.05)	(− 3.34)	(− 2.66)	(− 4.08)	(− 1.94)	(6.85)
Inverse Mills ratio	62.04***	67.68***	54.81***	72.74***	10.11***	− 0.198
	(24.08)	(23.31)	(23.14)	(18.44)	(3.52)	(− 0.62)
Constant	− 154.4***	− 175.2***	− 129.1***	− 154.9***	− 6.115	− 6.749***
	(− 10.96)	(− 11.09)	(− 9.88)	(− 7.23)	(− 0.38)	(− 5.46)
lns1_1_1 Constant	3.430***	3.406***	3.493***	3.655***	2.649***	0.271**
	(38.17)	(37.91)	(38.86)	(38.00)	(24.85)	(2.70)
lnsig_e Constant	3.324***	3.448***	3.237***	3.756***	3.485***	
	(308.85)	(320.48)	(300.61)	(348.83)	(324.29)	
Sector fixed effects	YES	YES	YES	YES	YES	YES
Lead-arranger fixed effects	YES	YES	YES	YES	YES	YES
Observations	4394	4394	4394	4394	4394	3406
Log-Likelihood	− 20962.6	− 21495.8	− 20588.9	− 22848.3	− 21611.1	− 578.8
Degrees of freedom	180	180	180	180	180	51

Standard errors in parenthesis; models 1–5 are multilevel mixed-effects linear regressions with project host-country random intercepts; model 6 is a multilevel mixed-effects probit regression with project host-country random intercepts

*p < 0.1; **p < 0.05; ***p < 0.01; *p* < 0.0001

**Table 4:** Random intercepts estimates with alternative measure of political risk

	Political leverage (H1)					IGO (H2)
	(7)	(8)	(9)	(10)	(11)	(12)
	Trade leverage	Export leverage	Import leverage	FDI leverage	Aid leverage	Intergov. organization
Political risk (PRS)	5.813***	6.754***	4.689***	6.078**	7.832***	0.327***
	**(4.11)**	**(4.31)**	**(3.56)**	**(2.88)**	**(5.75)**	**(4.03)**
Project size	12.85***	14.42***	10.89***	15.71***	0.517	0.312***
	(18.43)	(18.32)	(16.99)	(14.64)	(0.65)	(4.76)
Project with offtaker	7.834***	8.991***	6.603***	7.260***	0.0745	0.0342
	(6.04)	(6.13)	(5.55)	(3.63)	(0.05)	(0.26)
Debt-to-equity ratio	17.26***	19.84***	14.35***	20.45***	− 1.981	0.0670
	(6.82)	(6.93)	(6.18)	(5.23)	(− 0.67)	(0.27)
Systemic risk	0.368*	0.152	0.638***	− 1.157***	1.613***	0.907
	(2.43)	(0.89)	(4.60)	(− 4.96)	(9.09)	(1.51)
Total syndicate participants	− 0.0509	− 0.0582	− 0.0438	− 0.130**	− 0.102**	− 0.0817***
	(− 1.82)	(− 1.84)	(− 1.71)	(− 3.03)	(− 3.12)	(− 6.04)
Previous ties	− 0.243	− 0.332	− 0.150	− 0.373	0.665*	− 0.0122**
	(− 1.02)	(− 1.24)	(− 0.69)	(− 1.02)	(2.38)	(− 2.81)
Previous PF experience	− 1.092*	− 1.319**	− 0.889*	− 1.087	− 0.949	− 0.0253
	(− 2.41)	(− 2.58)	(− 2.14)	(− 1.56)	(− 1.82)	(− 0.96)
Banks’ local presence	− 1.145**	− 1.415***	− 0.917**	− 2.368***	− 0.816	− 0.0423
	(− 3.02)	(− 3.30)	(− 2.63)	(− 4.05)	(− 1.84)	(− 0.87)
No. of countries	− 25.08***	− 29.28***	− 19.92***	− 32.02***	− 6.623	0.226***
	(− 4.16)	(− 4.30)	(− 3.60)	(− 3.44)	(− 0.94)	(6.91)
Inverse Mills ratio	61.78***	67.40***	54.57***	72.27***	9.098**	− 0.246
	(23.94)	(23.18)	(22.99)	(18.26)	(3.14)	(− 0.76)
Constant	− 151.5***	− 172.7***	− 125.7***	− 148.9***	− 0.107	− 6.603***
	(− 10.75)	(− 10.93)	(− 9.61)	(− 6.94)	(− 0.01)	(− 5.36)
lns1_1_1 Constant	3.451***	3.429***	3.505***	3.659***	2.711***	0.290**
	(38.07)	(37.89)	(38.65)	(37.84)	(26.03)	(2.69)
lnsig_e Constant	3.325***	3.448***	3.238***	3.759***	3.488***	
	(308.85)	(320.41)	(300.72)	(349.01)	(324.51)	
Sector FE	YES	YES	YES	YES	YES	YES
Lead-Arranger FE	YES	YES	YES	YES	YES	YES
Observations	4392	4392	4392	4392	4392	3405
Log-Likelihood	− 20956.0	− 21486.5	− 20585.8	− 22847.8	− 21617.1	− 577.7
Degrees of freedom	180	180	180	180	180	51

Standard errors in parenthesis; models 1–5 are multilevel mixed-effects linear regressions with project host-country random intercepts; model 6 is a multilevel mixed-effects probit regression with project host-country random intercepts

*p < 0.1; **p < 0.05; ***p < 0.01; *p* < 0.0001

In model (1) in Table 3, a one-standard-deviation increase in socio-political risk in the project host country increases trade leverage by 7.465 percentage points (the equivalent of 0.2 standard deviations). Our model suggests that trade leverage would be 7.465 percentage points higher if the same project were financed in Tanzania instead of Romania (a change equivalent to a one-standard-deviation increase in socio-political risk), ceteris paribus.

Turning to the control variables, the coefficients are according to our expectations across the different measures of political leverage. Looking at model (1) in Table 3, we find that syndicate lead arrangers are more likely to form a partnership with high levels of political leverage when financing larger projects (*β* = 13.06; *p* = 0.000), projects with higher debt-to-equity ratios (*β* = 18.13; *p* = 0.000), and projects that have offtake agreements (often from the host-country government) (*β* = 7.704; *p* = 0.000). By contrast, systemic risk is negatively associated with the formation of global syndicates with high political risk (*β* = − 24.98; *p* = 0.000). Finally, the banks’ combined local presence is associated with lower political leverage at the syndicate level (*β* = − 1.208; *p* = 0.015) because local presence through subsidiary branches is likely a substitute for political leverage.

The analyses presented in model (6) in Table 3 and model (12) in Table 4 test Hypothesis 2 using mixed-effects probit models. Because some of the lead arrangers perfectly predict the presence of international organizations, our sample size is reduced to 3,406 and 3,405 projects, respectively. Consistent with our Hypothesis 2, the results in Table 3 indicate that the presence of intergovernmental organizations is significantly more likely (*β* = 0.317; *p* = 0.000 in Table 3) in syndicates that provide financing to projects in high-risk countries. A one-standard-deviation increase in socio-political risk increases the likelihood of the presence of an intergovernmental organization by 6% (when all other variables are held at their means). This is a sizable effect given a baseline probability of intergovernmental organizations of 10% in the full sample. The coefficient is very similar (*β* = 0.327; *p* = 0.000) when we re-run the analysis with the PRS measure of socio-political risk (model 6 in Table 4).

### Robustness Checks

To check the sensitivity of our results to different specifications, we re-ran the analyses with different model specifications, alternative measures of socio-political risk, and different calculations for our dependent variables of political leverage. In Table 5, we report the results using trade leverage as a dependent variable. Since the projects we analyze are nested in different countries, we test for the inclusion of project host-country dummies in models (13) and (14) to address the concern that unobserved characteristics of the host-country location may affect the results. The results similarly support our argument that syndicates have higher levels of political leverage when investing in countries with higher socio-political risk (*β* = 8.509; *p* = 0.000 in model 13). Our results also hold if we add host-country random coefficients to the random intercepts in our models. Results on the other measures of political leverage using imports, exports, FDI, or aid (not shown) are similarly robust, with *T*-statistics between 4.28 (*p* = 0.000) for export leverage and 6.2 (*p* = 0.000) for aid leverage. Similar to our baseline models, FDI leverage (*β* = 14.34) and aid leverage (*β* = 12.67) have the largest coefficients.

**Table 5:** Random intercepts estimates (alternative specifications)

	Political leverage (H1)					
	(13)	(14)	(15)	(16)	(17)	(18)
	Trade leverage	Trade leverage	Trade leverage	Trade leverage	Trade leverage	Trade leverage
Socio-political risk (EIU)	8.509***		8.632***		8.509***	
	**(4.90)**		**(4.83)**		**(4.75)**	
Socio-political risk (PRS)		6.745***		7.884***		6.745***
		**(4.47)**		**(5.09)**		**(4.34)**
Project size	13.54***	13.31***	1.072*	0.727	13.54***	13.31***
	(19.46)	(19.11)	(2.13)	(1.47)	(18.89)	(18.55)
Project with offtaker	7.883***	8.027***	2.703*	2.939*	7.883***	8.027***
	(6.12)	(6.23)	(2.04)	(2.26)	(5.94)	(6.05)
Debt-to-equity ratio	18.73***	17.74***	3.799	2.415	18.73***	17.74***
	(7.44)	(7.06)	(1.52)	(0.98)	(7.22)	(6.85)
Systemic risk	− 26.35***	− 26.43***	− 1.671	− 2.158	− 26.35***	− 26.43***
	(− 4.40)	(− 4.41)	(− 0.27)	(− 0.36)	(− 4.27)	(− 4.28)
Total syndicate participants	0.357*	0.363*	1.024***	1.059***	0.357*	0.363*
	(2.38)	(2.42)	(6.58)	(6.87)	(2.31)	(2.35)
Previous ties	− 0.0400	− 0.0547*	− 0.0386	− 0.0551	− 0.0400	− 0.0547
	(− 1.46)	(− 1.97)	(− 1.33)	(− 1.89)	(− 1.42)	(− 1.91)
Previous PF experience	− 0.200	− 0.247	0.152	0.125	− 0.200	− 0.247
	(− 0.85)	(− 1.05)	(0.63)	(0.52)	(− 0.82)	(− 1.02)
Banks’ local presence	− 0.993*	− 0.844	− 2.225***	− 1.923***	− 0.993*	− 0.844
	(− 2.20)	(− 1.87)	(− 4.61)	(− 4.13)	(− 2.13)	(− 1.81)
Nr. of countries	− 1.229**	− 1.218**	− 1.159**	− 1.181**	− 1.229**	− 1.218**
	(− 3.27)	(− 3.23)	(− 2.97)	(− 3.07)	(− 3.17)	(− 3.14)
Inverse Mills Ratio	64.48***	64.18***			64.48***	64.18***
	(24.92)	(24.76)			(24.18)	(24.04)
Constant	− 157.8***	13.31***	− 3.444	0.237	− 147.8***	− 148.6***
	(− 10.70)	(19.11)	(16.23)	(16.05)	(− 4.55)	(− 4.57)
lns1_1_1 Constant	− 11.51	8.027***	− 17.11***	− 15.96***		
	(− 0.08)	(6.23)	(2.351)	(2.080)		
lnsig_e Constant	3.315***	17.74***	3.398***	3.392***		
	(310.78)	(7.06)	(0.0103)	(0.0102)		
Sector FE	YES	YES	YES	YES	YES	YES
Lead-Arranger FE	YES	YES	YES	YES	NO	NO
Country FE	YES	YES	YES	YES	YES	YES
Selection Model	YES	YES	NO	NO	YES	YES
Observations	4394	4392	4746	4852	4394	4392
Log-Likelihood	− 20801.6	− 20795.1	− 22858.9	− 23341.1	− 22284.9	− 22275.7
Degrees of freedom	255	253	296	304	255	253

Standard errors in parenthesis; models 1–6 are multilevel mixed-effects linear regressions with project host-country random intercepts

*p < 0.1; **p < 0.05; ***p < 0.01; *p* < 0.0001

Second, in models (15) and (16), we show the results estimated without the inverse Mills correction estimated to approximate the probability of forming a syndicate with foreign banks. Results on trade leverage are significant (*β* = 8.632; *p* = 0.000 in model 3) for both measures of socio-political risk and are of comparable magnitude. Results on the other measures of leverage (not reported) are also significant (*p* = 0.000 throughout), with highest coefficients on FDI leverage (*β* = 17.12) and aid leverage (*β* = 13.25). *T*-statistics range from 4.22 for export leverage to 6.26 for aid leverage. Overall, our analysis is not sensitive to the inclusion or exclusion of the selection correction.

Third, in models (17) and (18), we run an OLS regression using lead-arranger, host-country, and industry fixed effects. Results remain highly significant (*β* = 8.509; *p* = 0.000 in model 5) and robust across all measures of political leverage (not reported), with *T*-statistics between 3.93 (*p* = 0.000) for export leverage and 8.14 (*p* = 0.000) for aid leverage. Again, coefficients on FDI leverage (*β* = 11.11) and aid leverage (*β* = 9.327) are highest.

Fourth, we argue in this paper that socio-political risk broadly defined affects the composition of banking partnerships financing large infrastructure projects. We intentionally selected measures constructed to capture a broader class of social and political events, including social unrest and political tensions at both national and subnational levels of government (which frequently affect politically sensitive infrastructure projects). In additional analyses, we also ran the models with measures of constraints on the host government using the Polcon (Henisz, 2000) and Polity (Marshall & Jaggers, 2000) measures. Unlike the EIU and PRS measures, which aim to capture a broad range of factors and events that influence socio-political risk, Polcon and Polity are designed to capture, in a narrower sense, the ability of the central government to unilaterally change government policies in ways that negatively affect investment such as the infrastructure projects in our dataset. When using either of these measures, we find partial support for Hypothesis 1, with a significant coefficient on aid leverage (*β* = 7.053; *p* = 0.000 in the specification using Polcon).

Fifth, because (a) the geographic location of a country naturally codetermines its socio-economic relationships with other countries (you trade more with your neighbors) and (b) because locations of banks that are realistic candidates for syndicates depend on the development of financial markets in particular countries, selection is the primary endogeneity concern in our empirical analysis. In Table A1 in the online appendix, we provide an instrumental-variable approach to the endogeneity problem. Because the location of the project codetermines trade dependence (a) used in the DV and the set of potentially available syndicate banks (b), we consider project location as endogenous and run a 2sls instrumental-variable regression with host-country political risk as the endogenous variable. We use the average geographic distance between syndicating banks as the first exogenous instrument. We argue that the distance between two banks (bank–bank) is independent of the trade imbalance between banks’ countries and the project country because it has no relationship to the project location. It is, however, theoretically related to political leverage, since banks will only syndicate across large distances if this distant partner can contribute a strategic value to the syndicate (home-bias). We also use a measure of the depth of the local financial market using World Bank data from Čihák et al. (2012) to capture the need for foreign banks within a project location as the second exogenous instrument. The results of instrumental 2SLS models provided in Table A3 in the online appendix support our conclusions for all models. Durbin Watson and Wu-Hausman tests confirms the endogeneity of location risk (*p* = 0.0000). The first-stage regression has an *F*-statistic of 1467.98 that is above the critical threshold of 19.93, indicating a moderately strong instrument. However, the partial *R*2 indicates a mediocre first-stage model fit (0.3843), and the Sargan and Basmann tests for overspecification are only narrowly insignificant (*p* = 0.0900).

## DISCUSSION AND CONCLUSION

Most broadly, our paper highlights that considerations of socio-political risk (and the institutional environment, more broadly) affect alliances and interorganizational partnerships. We propose that the degree of socio-political risk in a country, which reflects the probability that local stakeholders will behave opportunistically in ways that negatively affect a project’s successful completion and operation, creates incentives for organizations to think differently about the composition of their partnerships and to use the clout of non-local organizations to mitigate socio-political risk and to protect the project. We argue and demonstrate empirically that when financing projects in countries with high socio-political risk, syndicate lead arrangers are more likely to form banking partnerships that bring together banks from countries with high economic and political leverage over stakeholders in the host country and also more likely to include in the partnership intergovernmental organizations, which also provide political leverage.

At a theoretical level, our paper contributes three distinct elements to nonmarket strategy research in IB. First, we highlight the importance of theorizing on and measuring the political uncertainty of a particular country always with respect to its inter-country dependence relationships (i.e. supranational institutions). Our paper suggests that banks use loan syndication to pool economic dependencies that constrain potentially opportunistic host governments. Second, our theory and empirical results position non-local partners as a potentially more reliable alternative to local partners in countries with high political risk. Third, and finally, we hope to add a previously overseen perspective to the study of alliances as means of mitigating political risk. Specifically, we argue that in certain settings, debt-side creditors are better positioned to pool economic leverage over a host-country. In our paper, we document such leverage pooling in the context of large infrastructure projects and PF.

In his review of 2016, Jean Boddewyn refers to “a world where asymmetry reigns between a fairly integrated global economy and a rather fragmented international political order” and closes with a mandate for IB research to “focus on what MNE nonmarket strategies will fit these new circumstances” (p. 10). Since 2016, the global policy arena has become not only more disintegrated but also more volatile locally. Behind this backdrop of fragmented political order and political uncertainty, we argue that debt-side, non-local leverage can provide an alternative to many of the nonmarket strategies studied in IB.

In this paper, we use the empirical context of large infrastructure projects and PF. This empirical setting not only offers multiple advantages to researchers (discussed above); it also has immense practical importance in corporate practice, economic development, and the global policy agenda. The 2021 UNCTAD World Investment Report, for example, emphasizes the pivotal role of PF in sustainable recovery in the aftermath of COVID-19. Both academic research (Lundan & Leymann, 2021) and policy practice (Zhan et al., 2021) consider project investment to be among the most important avenues for future IB research. Therein, our study also fills a persistent empirical gap in IB literature.

There are several limitations to our research that point to potential avenues for future research. First, we can only observe pooling of political leverage, but we have no means of observing the effectiveness of debt-side leverage since there are no available data on equity or loan recovery from failing projects. Future research could compare equity- and debt-recovery rates after political expropriation and shed light on the relative strength and the contingencies of the two. Second, geographic location and economic activity are naturally interrelated. Just like individuals, countries tend to interact more with close countries. At the same time, financial markets and PF lenders are concentrated within a few developed countries. This creates an endogeneity between project location and political leverage from economic relationships. While we have made several attempts to account for this endogeneity, we cannot fully rule it out. Future research could collect additional data and develop more sophisticated research designs that allow for better control of endogeneity. Third, our data are focused on debt-side creditors. In a PF setting, these actors have an immensely important role in practice. Therefore, future IB and strategy research could initiate inquiries into the role of banks in MNE–state bargaining episodes. However, the role of these creditors in corporate (non-project finance) settings remains an unanswered question and an important boundary condition to date. Fourth, pooling of economic leverage – as observed in our setting – has natural boundary conditions related to the number of partners which can efficiently collaborate in a syndicate. Also, diversity of partners likely influences the costs of broad syndication. Finally, special types of partners like the Intergovernmental organizations studied in our paper by themselves constitute boundary conditions that moderate the observed theoretical mechanisms.

## Notes

1. Earlier work (e.g., Kobrin, 1979; Moran, 1973; Vernon, 1977) focuses on political risk defined as the risk that governments will take unilateral actions that negatively affect returns on multinational investments. Social risk (including social unrest, activist campaigns, and media attacks) has become more salient in recent decades, and needs to be considered alongside political risk when discussing local risks for multinational investments. We use the term *socio-political risk* interchangeably with the more established term *political risk* to refer to both political and social events that can result in the appropriation of value created by the multinational investment in excess of contractual agreements.
2. Given the nature of PF, in which the project company is the borrower and there is no legal recourse for equity providers behind the project, the political risk faced by projects is also borne by the projects’ creditors. Non-recourse means that creditors cannot reclaim loans from equity sponsors of the project. If the project cannot meet its financial obligations and defaults, the only legal remedy for creditors is the intervening host government. Hence, project finance borrowers face political risk.
3. In an analysis of investment disputes using data from the International Center for Settlement of Disputes, Caddel and Jensen (2014) showed that more than half of international investment disputes were filed against executive and legislative bodies who allegedly changed the terms of the investment *ex post*.
4. Data coverage is comparable to rating agencies such as Moody’s, which recorded 6595 projects over the period and claims to have historic coverage of over 70% since 1983 (Moody’s, 2015).
5. This distribution is consistent with industry league tables classifying the activity of banks in project finance (available from Thomson Reuters and Bloomberg).
6. For most cases, the syndicate-level measure is between − 100 and + 100. Because some syndicates have two or more banks from the same home country, the aggregate value can exceed 100%.
7. Because our measure of socio-political risk is standardized, the marginal effect calculated at means is equal to the regression coefficient (β = 7.452).

## Supplementary Information

Below is the link to the electronic supplementary material.

Supplementary material 1 (DOCX 33 kb)
