# Supplementary material for: Overcoming political risk in developing economies through non-local debt
Source: J Int Bus Policy. 2022 May 3;6(2):159–81. doi: 10.1057/s42214-022-00137-w (PMC9062290; doi:10.1057/s42214-022-00137-w)
Supplement: Supplementary file 1 — Supplementary material 1 (DOCX 33 kb) [file 42214_2022_137_MOESM1_ESM.docx]

**Appendix A. Geographic and temporal distribution of projects.**

| Project country /year | 2000 | 2001 | 2002 | 2003 | 2004 | 2005 | 2006 | 2007 | 2008 | 2009 | 2010 | 2011 | 2012 | Total |
| --- | --- | --- | --- | --- | --- | --- | --- | --- | --- | --- | --- | --- | --- | --- |
| United States | 85 | 52 | 27 | 35 | 45 | 51 | 79 | 73 | 71 | 68 | 78 | 81 | 78 | 823 |
| United Kingdom | 45 | 47 | 49 | 53 | 73 | 59 | 59 | 59 | 27 | 30 | 39 | 43 | 32 | 615 |
| Spain | 16 | 15 | 8 | 17 | 26 | 34 | 41 | 50 | 96 | 76 | 83 | 62 | 29 | 553 |
| Australia | 35 | 17 | 26 | 43 | 32 | 51 | 47 | 34 | 43 | 29 | 32 | 57 | 45 | 491 |
| India | 3 | 1 | 2 | 3 | 3 | 3 | 4 | 8 | 18 | 28 | 71 | 92 | 38 | 274 |
| Brazil | 29 | 23 | 6 | 10 | 12 | 16 | 13 | 16 | 15 | 29 | 16 | 25 | 23 | 233 |
| Italy | 10 | 8 | 4 | 11 | 3 | 22 | 7 | 18 | 15 | 21 | 33 | 24 | 9 | 185 |
| France | 5 | 3 | 5 | 5 | 0 | 10 | 15 | 25 | 10 | 9 | 22 | 26 | 29 | 164 |
| Canada | 2 | 5 | 4 | 1 | 6 | 5 | 11 | 17 | 12 | 17 | 30 | 21 | 19 | 150 |
| South Korea | 2 | 3 | 0 | 3 | 6 | 10 | 9 | 25 | 13 | 11 | 15 | 11 | 14 | 122 |
| Mexico | 14 | 7 | 7 | 6 | 10 | 6 | 12 | 4 | 10 | 7 | 13 | 8 | 14 | 118 |
| Chile | 5 | 8 | 9 | 10 | 13 | 10 | 4 | 10 | 5 | 6 | 3 | 4 | 8 | 95 |
| Germany | 9 | 3 | 1 | 1 | 9 | 7 | 6 | 8 | 8 | 6 | 12 | 15 | 4 | 89 |
| Indonesia | 2 | 0 | 0 | 7 | 6 | 9 | 7 | 6 | 10 | 15 | 8 | 9 | 10 | 89 |
| China | 4 | 0 | 5 | 5 | 4 | 4 | 4 | 8 | 4 | 15 | 13 | 10 | 10 | 86 |
| Japan | 1 | 4 | 4 | 5 | 5 | 13 | 11 | 6 | 8 | 8 | 4 | 3 | 5 | 77 |
| Portugal | 4 | 6 | 2 | 3 | 6 | 7 | 5 | 7 | 15 | 6 | 8 | 5 | 0 | 74 |
| Russian Federation | 4 | 2 | 1 | 1 | 4 | 2 | 1 | 6 | 4 | 9 | 12 | 10 | 1 | 57 |
| Turkey | 6 | 0 | 2 | 0 | 0 | 3 | 2 | 7 | 7 | 4 | 4 | 11 | 7 | 53 |
| Saudi Arabia | 5 | 2 | 3 | 3 | 5 | 1 | 4 | 10 | 5 | 2 | 5 | 5 | 2 | 52 |
| Thailand | 6 | 1 | 5 | 2 | 4 | 4 | 3 | 4 | 3 | 1 | 6 | 7 | 6 | 52 |
| Poland | 4 | 6 | 1 | 1 | 2 | 2 | 3 | 1 | 10 | 3 | 7 | 9 | 1 | 50 |
| Peru | 0 | 2 | 3 | 1 | 3 | 5 | 4 | 2 | 4 | 6 | 5 | 8 | 6 | 49 |
| Ireland | 1 | 3 | 1 | 5 | 3 | 4 | 7 | 5 | 2 | 6 | 7 | 2 | 2 | 48 |
| Qatar | 3 | 2 | 2 | 2 | 4 | 10 | 4 | 5 | 5 | 5 | 0 | 1 | 2 | 45 |
| Singapore | 4 | 2 | 2 | 0 | 0 | 2 | 4 | 6 | 4 | 5 | 1 | 7 | 6 | 43 |
| Malaysia | 4 | 4 | 3 | 3 | 1 | 2 | 2 | 1 | 3 | 2 | 2 | 3 | 12 | 42 |
| United Arab Emirates | 2 | 0 | 2 | 2 | 1 | 2 | 4 | 4 | 5 | 11 | 3 | 3 | 3 | 42 |
| Philippines | 5 | 5 | 1 | 1 | 2 | 1 | 5 | 3 | 7 | 3 | 3 | 1 | 3 | 40 |
| Nigeria | 0 | 1 | 2 | 3 | 4 | 3 | 2 | 3 | 3 | 7 | 5 | 5 | 1 | 39 |
| Oman | 1 | 2 | 3 | 2 | 4 | 10 | 6 | 4 | 1 | 1 | 2 | 2 | 1 | 39 |
| Vietnam | 0 | 1 | 1 | 1 | 1 | 1 | 2 | 2 | 3 | 5 | 3 | 12 | 6 | 38 |
| Belgium | 2 | 1 | 1 | 0 | 1 | 2 | 0 | 1 | 5 | 7 | 7 | 7 | 3 | 37 |
| South Africa | 2 | 6 | 1 | 2 | 1 | 2 | 3 | 0 | 1 | 9 | 5 | 2 | 3 | 37 |
| Netherlands | 5 | 0 | 2 | 2 | 2 | 0 | 2 | 2 | 6 | 4 | 5 | 4 | 2 | 36 |
| Argentina | 11 | 9 | 0 | 2 | 3 | 0 | 1 | 4 | 2 | 0 | 1 | 0 | 1 | 34 |
| Greece | 4 | 0 | 1 | 2 | 2 | 1 | 3 | 2 | 9 | 4 | 5 | 0 | 0 | 33 |
| Egypt | 1 | 2 | 0 | 0 | 2 | 3 | 1 | 4 | 5 | 3 | 5 | 1 | 1 | 28 |
| Colombia | 5 | 3 | 1 | 1 | 2 | 2 | 0 | 2 | 1 | 2 | 2 | 2 | 4 | 27 |
| New Zealand | 3 | 0 | 1 | 1 | 1 | 2 | 6 | 2 | 1 | 0 | 0 | 1 | 6 | 24 |
| Panama | 1 | 1 | 1 | 2 | 0 | 2 | 0 | 2 | 4 | 2 | 3 | 3 | 2 | 23 |
| Hungary | 3 | 2 | 1 | 4 | 2 | 5 | 1 | 1 | 2 | 0 | 1 | 0 | 0 | 22 |
| Ghana | 0 | 0 | 0 | 0 | 1 | 2 | 1 | 0 | 0 | 6 | 3 | 5 | 3 | 21 |
| Czech Republic | 2 | 3 | 0 | 1 | 1 | 1 | 1 | 0 | 0 | 1 | 7 | 2 | 0 | 19 |
| Bulgaria | 2 | 0 | 0 | 1 | 3 | 1 | 2 | 1 | 1 | 1 | 1 | 2 | 3 | 18 |
| Dominican Republic | 1 | 0 | 4 | 2 | 0 | 2 | 1 | 1 | 1 | 0 | 5 | 0 | 1 | 18 |
| Hong Kong | 6 | 3 | 3 | 0 | 0 | 2 | 2 | 0 | 1 | 0 | 1 | 0 | 0 | 18 |
| Kenya | 1 | 1 | 1 | 0 | 2 | 1 | 2 | 0 | 1 | 4 | 0 | 2 | 3 | 18 |
| Papua New Guinea | 1 | 1 | 1 | 2 | 1 | 1 | 1 | 2 | 2 | 2 | 1 | 1 | 2 | 18 |
| Taiwan | 3 | 3 | 1 | 2 | 1 | 1 | 0 | 0 | 2 | 1 | 1 | 2 | 1 | 18 |
| Trinidad and Tobago | 2 | 0 | 3 | 1 | 2 | 0 | 6 | 4 | 0 | 0 | 0 | 0 | 0 | 18 |
| Pakistan | 0 | 0 | 0 | 3 | 1 | 0 | 0 | 1 | 1 | 2 | 2 | 3 | 2 | 15 |
| Romania | 0 | 1 | 3 | 0 | 1 | 3 | 1 | 0 | 0 | 2 | 0 | 3 | 1 | 15 |
| Venezuela | 2 | 1 | 1 | 1 | 1 | 2 | 1 | 1 | 2 | 1 | 0 | 2 | 0 | 15 |
| Algeria | 2 | 0 | 1 | 2 | 3 | 2 | 0 | 2 | 1 | 1 | 0 | 0 | 0 | 14 |
| Croatia | 0 | 2 | 1 | 2 | 1 | 0 | 0 | 0 | 3 | 2 | 1 | 1 | 1 | 14 |
| Finland | 1 | 0 | 0 | 0 | 1 | 1 | 0 | 1 | 0 | 2 | 2 | 4 | 2 | 14 |
| Norway | 1 | 0 | 0 | 1 | 1 | 0 | 1 | 2 | 0 | 1 | 3 | 2 | 2 | 14 |
| Zambia | 3 | 0 | 0 | 1 | 0 | 0 | 0 | 2 | 2 | 2 | 2 | 0 | 2 | 14 |
| Total | 418 | 298 | 240 | 303 | 368 | 432 | 444 | 498 | 531 | 552 | 647 | 691 | 506 | 5928 |

Continued: 13 Jamaica, Laos, Sweden 12 Guatemala, Macau 11 Mozambique, 10 Angola, Bahrain, Cameroon, Denmark, Israel, Jordan, 9 Bahamas, 8 Bolivia, Cote d'Ivoire, Kazakhstan, Morocco, Tanzania, 7 Azerbaijan, Iran, Senegal, Slovak Republic, Switzerland, 6 Uganda, Congo, Costa Rica, Estonia, Gabon, Ukraine, 5 Honduras, Iceland, Kuwait, Nicaragua, Sierra Leone, Slovenia, Yemen, 4 Armenia, Austria, Bangladesh, Botswana, Cyprus, Lithuania, Netherlands Antilles, Tunisia, Uruguay, Uzbekistan, 3 Brunei, Cape Verde Islands, Ecuador, El Salvador, Guadeloupe, Guinea, Haiti, Sri Lanka, Sudan, 2 Afghanistan, Albania, Bosnia-Herzegovina, Burkina Faso, Ethiopia, Georgia, Latvia, Lebanon, Mali, Mauritania, Mauritius, Namibia, New Caledonia, Puerto Rico, Turkmenistan, Zimbabwe, 1 Andorra, Aruba, Benin, Bermuda, Bhutan, Cambodia, Cayman Islands, Chad, Djibouti, Equatorial Guinea, Eritrea, Fiji, Iraq, Kyrgyz Republic, Libya, Macedonia, Madagascar, Malawi, Monaco, Mongolia, Montenegro, Myanmar, Nepal, Niger, Palestine, Rwanda, Seychelles, Syria, Togo, Turks & Caicos

**Appendix B. Distribution of projects across industries.**

| Project sector | N | Percent |
| --- | --- | --- |
| Power | 1,126 | 18.99 |
| Wind farm | 661 | 11.15 |
| Renewable fuel | 602 | 10.16 |
| Road | 428 | 7.22 |
| Mining | 326 | 5.5 |
| Telecom | 293 | 4.94 |
| Education | 217 | 3.66 |
| Petrochemical/Chemical plant | 214 | 3.61 |
| Oilfield exploration and development | 206 | 3.48 |
| Hospital | 198 | 3.34 |
| Oil Refinery / LNG | 195 | 3.29 |
| Water and sewerage | 140 | 2.36 |
| Airport | 124 | 2.09 |
| Gas pipeline | 116 | 1.96 |
| Port | 116 | 1.96 |
| Processing plant | 85 | 1.43 |
| Gasfield exploration and development | 84 | 1.42 |
| Government buildings | 74 | 1.25 |
| Other infrastructure projects | 69 | 1.16 |
| Rail - infrastructure | 69 | 1.16 |
| Gas distribution | 64 | 1.08 |
| Waste | 62 | 1.05 |
| Urban railway / LRT | 53 | 0.89 |
| Recreational facilities | 45 | 0.76 |
| Steel mill | 45 | 0.76 |
| Hotel/resort/casino | 36 | 0.61 |
| Defense | 35 | 0.59 |
| Prison | 33 | 0.56 |
| Other upstream | 26 | 0.44 |
| Tunnel | 24 | 0.40 |
| Oil pipeline | 21 | 0.35 |
| Commercial property | 20 | 0.34 |
| Residential property | 20 | 0.34 |
| Other* | 101 | 1.70 |
| Total | 5,928 | 100 |

*Other: 18 Manufacturing facilities, 17 Bridges, 16 Police facilities, 14 Pulp & paper, 10 Telecommunications facilities, 8 Industrial/Commercial zones, 6 other downstream oil/gas facilities, 6 Shipping, 2 Aircraft, Project equipment, 1 Agricultural, Rail equipment.

**Appendix C. Instrumental regressions**

|  | (1) | (2) | (3) | (4) | (5) | (6) |
| --- | --- | --- | --- | --- | --- | --- |
|  | Trade leverage | Export leverage | Import leverage | FDI leverage | Aid  leverage | Intergov. organization |
| **Socio-political risk (EIU)** | 27.26*** | 32.40*** | 21.39*** | 44.53*** | 7.349*** | 0.0570*** |
|  | (21.96) | (23.26) | (19.40) | (25.05) | (8.16) | (8.64) |
| Project size | 1.249* | 1.367* | 1.017* | 1.686* | -0.191 | 0.0156*** |
|  | (2.20) | (2.14) | (2.02) | (2.07) | (-0.46) | (5.17) |
| Project with offtaker | -5.897*** | -6.536*** | -5.121*** | -11.59*** | -0.121 | -0.00611 |
|  | (-3.44) | (-3.40) | (-3.37) | (-4.73) | (-0.10) | (-0.67) |
| Debt-to-equity ratio | 13.41*** | 15.85*** | 10.57*** | 17.21*** | 1.685 | -0.0207 |
|  | (4.50) | (4.74) | (4.00) | (4.04) | (0.78) | (-1.31) |
| Systemic risk | 7.347 | 9.209 | 4.556 | -13.07 | 26.32** | 0.0869 |
|  | (0.52) | (0.58) | (0.36) | (-0.65) | (2.58) | (1.16) |
| Total syndicate participants | -0.274 | -0.483* | 0.00987 | -1.757*** | 0.347** | -0.00889*** |
|  | (-1.51) | (-2.37) | (0.06) | (-6.75) | (2.63) | (-9.22) |
| Previous ties | 0.337 | 0.0874 | 0.591 | -2.447*** | 1.146*** | 0.0288*** |
|  | (0.79) | (0.18) | (1.55) | (-3.98) | (3.68) | (12.62) |
| Previous PF experience | 0.0170 | 0.0138 | 0.0201 | -0.106 | -0.00206 | -0.000560** |
|  | (0.44) | (0.32) | (0.59) | (-1.93) | (-0.07) | (-2.74) |
| Banks’ local presence | 1.593*** | 1.651*** | 1.530*** | 1.274** | 0.460* | -0.00513*** |
|  | (5.70) | (5.27) | (6.17) | (3.18) | (2.27) | (-3.46) |
| Nr. of countries | -1.960*** | -1.566** | -2.348*** | 2.145** | -1.343*** | -0.00158 |
|  | (-4.03) | (-2.87) | (-5.44) | (3.08) | (-3.81) | (-0.61) |
| Constant | -17.63* | -23.02* | -10.34 | -35.63** | 2.601 | -0.198*** |
|  | (-2.11) | (-2.45) | (-1.39) | (-2.97) | (0.43) | (-4.46) |
| Instruments | YES | YES | YES | YES | YES | YES |
| Year FE | YES | YES | YES | YES | YES | YES |
| Industry FE | YES | YES | YES | YES | YES | YES |
| Observations | 4730 | 4730 | 4730 | 4730 | 4730 | 4730 |
| chi2 | 863.4 | 902.5 | 788.4 | 1102.0 | 336.1 | 445.1 |
| df_m | 26 | 26 | 26 | 26 | 26 | 26 |

We consider project location as endogenous and run a 2sls instrumental variable regression with host-country political risk as the endogenous variable. We use the average geographic distance between syndicating banks and host-country financial market depth as exogenous instruments. Durbin Watson and Wu-Hausman tests confirms the endogeneity of location risk (p=0.0000). The first stage regression has an F-statistic of 1467.98 above the critical threshold of 19.93. Partial R2 indicates first stage model fit of 0.3843 and the Sargan & Basmann tests for overspecification is narrowly insignificant (p= 0.0900).
